# Time Course Changes of the Mechanical Properties of the Iris Pigment Epithelium in a Rat Chronic Ocular Hypertension Model

**DOI:** 10.1155/2018/4862309

**Published:** 2018-10-21

**Authors:** Tan Li, Lin Li, Zhicheng Liu

**Affiliations:** ^1^School of Biomedical Engineering, Capital Medical University, Beijing 100069, China; ^2^Beijing Key Laboratory of Fundamental Research on Biomechanics in Clinical Application, Capital Medical University, Beijing 100069, China

## Abstract

**Background:**

The flow field of aqueous humor correlates to the stiffness of iris pigment epithelium (IPE) which acts as a wall of posterior chamber. We focus on the variations of IPE stiffness in a rat ocular hypertension (OHT) model, so as to prepare for exploring the mechanism of duration of OHT.

**Methods:**

Episcleral venous cauterization (EVC) was applied on one eye of male adult Sprague-Dawley rats to induce chronic high intraocular pressure. According to the duration of OHT (0, 1, 2, 4, and 8 weeks), rats were randomly divided into Gw0, Gw1, Gw2, Gw4, and Gw8. Atomic force microscope (AFM) analysis was applied to test IPE stiffness in three regions: iris root, mid-periphery, and pupillary-margin in each group. Histological changes of IPE were also examined in Gw4 and Gw8.

**Results:**

There was an overall growing tendency of IPE stiffness in EVC eye. IPE in EVC eye was significantly stiffer than fellow eye in Gw2, Gw4, and Gw8 (in iris root, mid-periphery, and pupillary-margin, p<0.05). IPE in EVC eye in pupillary-margin was significantly stiffer than iris root in Gw4 and Gw8 (p<0.05). In EVC eye, IPE becomes thinner and IPE cell density decreases.

**Conclusion:**

IPE stiffness increases gradually with the duration of chronic high intraocular pressure.

## 1. Introduction

Persistent high intraocular pressure (IOP) has been regarded as the main clinical manifestation of glaucoma which is the first leading cause of irreparable blindness worldwide [1]. Physiologically, aqueous humor (AH) of the eye is secreted by the ciliary process, flows through posterior chamber and pupil, enters anterior chamber, and exits the eye via the trabecular meshwork at last. The flow of AH is affected by multiple factors, such as pupillary blocking force [2–5], wall shear stress [6], and the resistance to AH outflow through the trabecular pathway [7, 8]. Pathological changes of any factor may cause abnormal flow of AH and elevate intraocular pressure, which may lead to ocular hypertension (OHT). In fact, the pressure drop from posterior chamber to anterior chamber drives AH flow. And posterior chamber is a narrow iris-lens double wall channel: one wall is iris pigment epithelium (IPE) and the other is combined with lens and zonule. A recent publication focuses on the flow of aqueous humor in the posterior chamber [9]. In that study, a mathematical model of aqueous motion in the posterior chamber has been developed and the results indicate that the volume change of posterior chamber has an appreciable impact on the AH flow, even as small as 5%. Therefore we think that more attentions should be paid to the flow of AH in the posterior chamber. However, little research on AH flow in the posterior chamber has been reported.

According to the theory of fluid dynamics, the flow field is directly related to stiffness of the surrounding tissues and a number of biomechanical researches have also demonstrated this point [10–12]. So we wonder how the IPE stiffness affects the flow of AH in the posterior chamber. Prior to this, IPE stiffness is necessary as a basic data.

In fact, dating back to 1985, Shimizu and Futa [13] found a good correlation between the glaucoma stage and changes of ultrastructural characterization of IPE. In stage 1 of the duration of glaucoma, an increase in the amount of infolding appears in the IPE; in stage 2, IPE cells gradually disintegrate and the basement membrane increases in thickness; in stage 3, marked degeneration and disintegration combined with discontinuous thick basement membrane are still found in the IPE. The increased thickness of basement membrane inspires us that IPE may be stiffer in the course of glaucoma, for the reason that basement membrane is full of type IV collagen which may play a key role in mechanistic level [14, 15].

Many researches have shown that pathogenesis of glaucoma is closely related to the duration of high IOP [16–18]. Our hypothesis is as follows: on one hand, remodeling of IPE maybe happens under chronic high intraocular, which is manifested as the changes of IPE stiffness and other characteristics of IPE. On the other hand, the change of IPE stiffness may affect the flow of AH in turn from mechanical point of view. Perhaps this change leads to a variation of the characteristics of IPE, such as shedding of IPE. Either the excess of pigment that is not well disposed by endothelial cells of the trabecular meshwork or abnormal flow field in the posterior chamber may result in the resistance to AH outflow through the trabecular pathway. It would lead to elevation of IOP and then high IOP sustains. In fact, the mechanical properties, structures, and functions of living tissues generally interact with each other [19].

In this study, we are mainly trying to verify the first half of our hypothesis: IPE stiffness may change under chronic high intraocular pressure. First of all, IOP elevation was induced by episcleral venous cauterization (EVC) on rat eyes. Subsequently, at different time points (presurgery; 1st, 2nd, 4th, and 8th weekend after OHT induction), the IPE stiffness was measured by atomic force microscope (AFM) indentation tests, which has powerful ability to probe mechanical properties of ocular tissues including the stiffness of iris and trabecular meshwork in glaucomatous donors [20, 21]. Histology analysis was also performed to explore whether the integrity of IPE was damaged at 4th and 8th weekend after OHT induction. At last, IPE stiffness under different duration of OHT was obtained.

## 2. Materials and Methods

### 2.1. Animals

All experiments were complied with relevant laws and institutional regulations. Male adult Sprague-Dawley rats (7-8 weeks, 290-300 g weight) were obtained from the Experimental Animal Department of the Capital Medical University and given at least 48 hours of adaptation time. They were housed libitum and maintained in an air-conditioned room in a 12-h light/12-h dark cycle. All surgeries were performed under systemic anesthesia, and all efforts were made to minimize suffering.

A total of 40 rats were involved in this experiment. According to different duration of OHT, they were divided into 5 groups, denoted by Gw0 which was executed before OHT induction and Gw1, Gw2, Gw4, and Gw8 which were executed at 1st, 2nd, 4th, and 8th weekend after OHT induction, respectively.  Table 1 shows the number of each kind of rat prepared for indentation in each group. There were also 5 rats prepared for histology which were not included in Table 1. In order to save the use of experimental animals, left eye and right eye of Blank-control rat in Gw0 were regarded as the same.

### 2.2. Episcleral Venous Cauterization

In this study, the method of OHT induction was based mainly on the research of Mittag [22]. Rats were anesthetic by 1% sodium pentobarbital with the dosage of 0.4ml per 100 g weight. Incision of the conjunctiva exposed two dorsal episcleral veins located near the superior rectus and temporal episcleral vein near the lateral rectus. Then the veins were lift up and cauterized with an ophthalmic cautery carefully. Levofloxacin eye drops (Santen, Osaka, Japan) were used to diminish inflammation. After the conjunctival incision had healed (4-5 days after cauterization), a subconjunctival injection of 100*μ*L of 5-fluorouracil (5-Fu, Haipu Pharmaceutical Co., Shanghai, China) with a concentration of 25mg/mL was also performed by a 29G needle on the EVC eyes. A week after the first injection of 5-Fu, a subconjunctival injection of 100*μ*L of 5-Fu was performed again (only including Gw2, Gw4, and Gw8, because Gw1 had been executed at that time). Sham-operation eyes (SHO eyes) were sham-operated by receiving conjunctival incision and injection of 5-Fu at the same time with EVC eyes but without cauterization. All kinds of Blank-control eyes (BC-I eye, BC-II eye, and BC-III eye) received no treatment.

### 2.3. IOP Measurements

The IOP of the rats was measured with a TonoLab Rebound Tonometer (Icare, Vantaa, Finland) at presurgery, half a week, and 1, 2, 3, 4, 6, and 8 week(s) after OHT induction as long as the rat had not been executed. In order to avoid the effect of circadian rhythm, IOP measurements were arranged between 10 AM and 12AM on the awakened rats. If the range of the IOP among three times of measurements was less than 3mmHg, the median would be regarded as an effective value.

### 2.4. Histology

Presurgery and at 4th and 8th weekend after OHT induction, the rats prepared for histology were executed by excessive amounts of pentobarbital. A pair of eye balls was completely removed as soon as possible. The eyes were fixed with eye fixation fluid for about 24h. Then the eyes were dehydrated by graded ethanol and embedded by paraffin. The resin was cut into 3*μ*m thin sections along the sagittal direction of the eye and processed with Masson trichrome stain. A total of 20 histological cross-sections per iris were performed. Histological cross-sections were examined by a ZEISS Axio Lab A1 light microscope (Carl Zeiss, Jena, Germany).

### 2.5. Iris Specimen Preparation for Indentation

**Figure 1:**
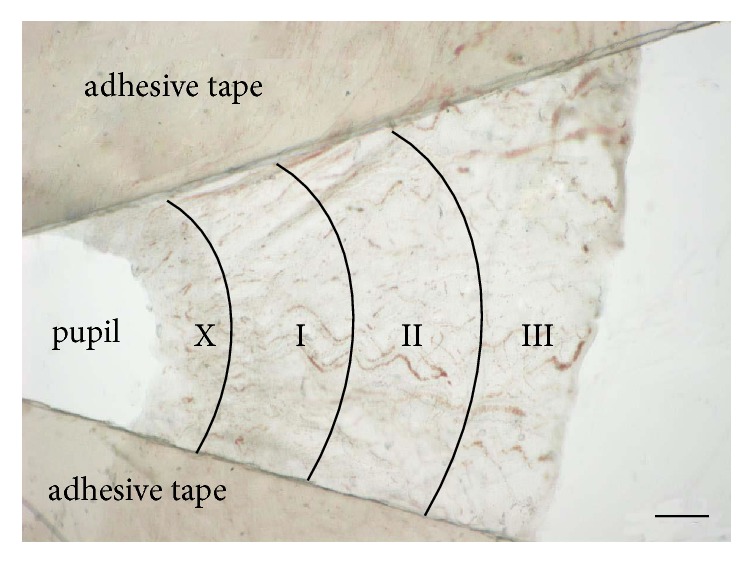
A performed iris sample prepared for indentation. The iris was partitioned along the radial direction. X: iris sphincter muscle region which was not indented. I: pupillary-margin. II: mid-periphery. III: iris root. Bar=200*μ*m.

Rats were executed by injection of excess pentobarbital. Then both of the eyes were removed immediately. Each of them was put into a Petri dish (60mm×15mm) full of phosphate buffered saline (PBS). The eyeball was cut open through the sclera with micro scalpel for ophthalmic surgery. Lens and vitreous were removed. The whole circle of limbus was cut open precisely by an iris forceps, with the iris separated from the eyeball wall naturally. Then a pipette was used to suck out all of the PBS in the culture dish, keeping the iris flat. Residual PBS was dried completely by napkin without touching the iris. Adhesive tape (Scotch®) was cut in the middle to make a small hole. It was used to fix the iris on the dish with the pigment epithelium facing up. A sector field of the specimen was exposed for indentation. Finally, the dish was filled with PBS to keep the tissue alive. A performed iris sample is shown in Figure 1.

### 2.6. Indentation

The iris sample in the PBS was placed under an AFM (NTEGRA, NT-MDT, Russia). We adopted a spherical probe (MLCT-O10-A, Bruker) stuck to the tip of the cantilever; and its radius (**r**) was 10*μ*m. Loading speed was 2*μ*m/s.

**Figure 2:**
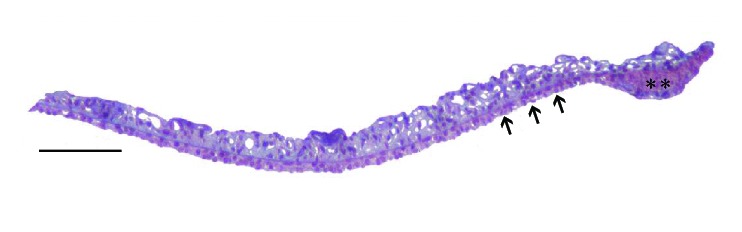
Iris histological cross-section of a Blank-control rat in Gw0. The arrow points to IPE, which shows a typical two-layer construction. The position of black ‘*∗∗*' is iris sphincter muscle. Bar=100*μ*m.

**Figure 3:**
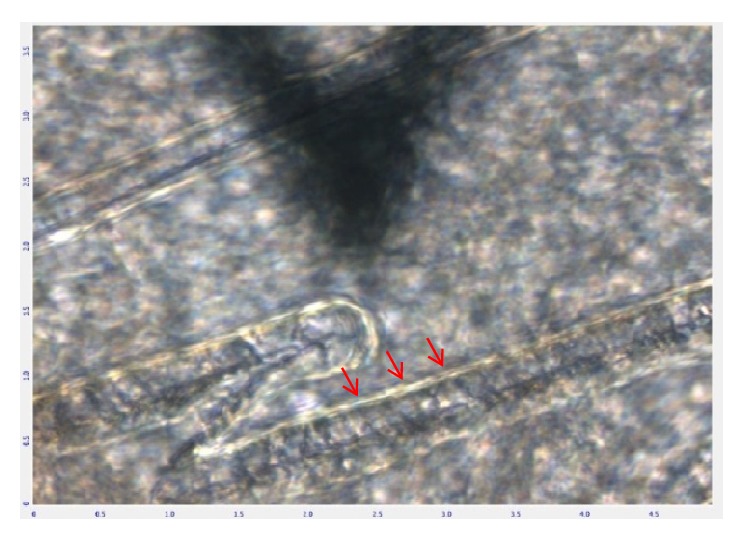
Image of AFM probe on the iris of a rat captured by the inverted microscope. The indentation points got away from the blood vessel which was shown by red arrows.

According to Figure 2, an iris histological cross-section of a Blank-control rat in Gw0, the indentation points avoided the sphincter muscle part of the iris with a width of 200*μ*m near the pupil for the reason that IPE was very thin or even missing in this region. As shown in Figure 1, remaining part was evenly divided into three regions: iris root, mid-periphery, and pupillary-margin by a reference coordinate system in the AFM. The indentation points were set along the radial direction of the iris, avoiding blood vessels in the stroma shown in Figure 3, a typical image of the iris taken optically by the inverted microscope of the AFM.

Although the AFM had a labeled spring constant of 0.05N·m^−1^, we determined its actual spring constant (**k**) prior to the first test on each experiment day. During tissue indentation, piezo-actuator translation (**Z**) and the cantilever deflection (**d**) were measured primordially. Then they were used to calculate the indentation force (**F**) and **w**, where **F** = **k***∗ ***d**, **w** = **Z** − **d**. The relation between **F** and **w** could be described by Hertz equation:

(1) $$\begin{array}{c} F=\frac{4}{3}\frac{r^{1/2}{\left(w-a\right)}^{3/2}}{1-\nu^{2}}E+b \end{array}$$

where ***ν*** is Poisson's ratio; it was assumed to be 0.5 according to previous studies [23]. **E** is the elastic modulus of IPE which we wanted to get finally. (**a**, **b**) is the contact point which would be found automatically during the process of regression analysis.

It is necessary to point out that, before regression analysis, the upper and lower limits of fitting range need to be selected artificially. As shown in Figure 7, the contact point was chosen as the lower limit of the fitting range and its abscissa was recorded as **w**_**c**_. For the reason that Hertz equation requires tissues to be homogeneous and linear elastic [24, 25], infinitesimal deformation of the tissue was necessary. Furthermore, it required **δ** (the depth of indentation) <0.1**r**. This law guided us to choose the upper limit of the fitting range.

During indentation experiments, if a **Z**-**d** curve was irregular or the contact point could hardly be found on the curve, we would choose another indentation point nearby. There were three indentation curves performed in each region. Then two curves with higher R^2^-values from these three curves were selected as effective curves to calculate the stiffness. R^2^-values in all effective curves were observed to be greater than 0.9.

### 2.7. Statistical Analyses

One-way ANOVA followed by post hoc Tukey testing was applied to compare IPE stiffness within each group except Gw0 by GraphPad Prism (GraphPad Software, Inc., La Jolla, CA).* p*< 0.05 indicated a statistically significant difference.

## 3. Results

### 3.1. Intraocular Pressures in Episcleral Venous Cauterization Model

**Figure 4:**
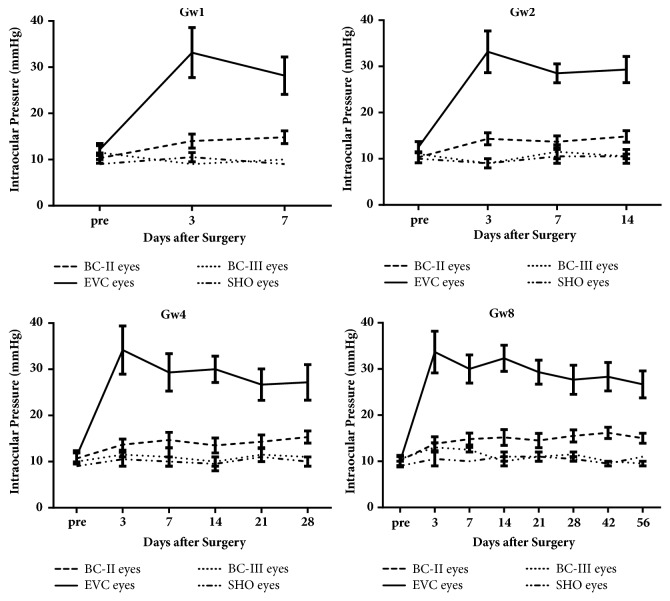
The IOP of rats in Gw1, Gw2, Gw4, and Gw8. Episcleral venous cauterization eyes (EVC eyes) and Blank control-II eyes (BC-II eyes) come from OHT rats; sham-operation eyes (SHO eyes) and Blank control-III eyes (BC-III eyes) come from sham-operation rats. Data are shown as mean±sd.

Figure 4 shows the IOP of OHT rats (including EVC eyes and BC-II eyes) and sham-operation rats (including SHO eyes and BC-III eyes) in Gw1, Gw2, Gw4, and Gw8. At 3rd day after the first time of cauterization, IOP of EVC eyes increased to 30-40 mmHg. Then it decreased slightly, but remained above 25mmHg until the end of the experiment. There was also a smaller increase in IOP of the BC-II eyes. SHO eyes and BC-III eyes of sham-operation rats in Gw1, Gw2, Gw4, and Gw8 had no significant changes. The IOP of BC-I eyes of Gw0 which was executed without OHT induction was 9.3±0.6mmHg (not shown in Figure 4).

### 3.2. Histology

By examining all the slices, we verified the continuity and uniformity of IPE distribution in EVC eyes at 4^th^ and 8^th^ week after OHT induction.

**Figure 5:**
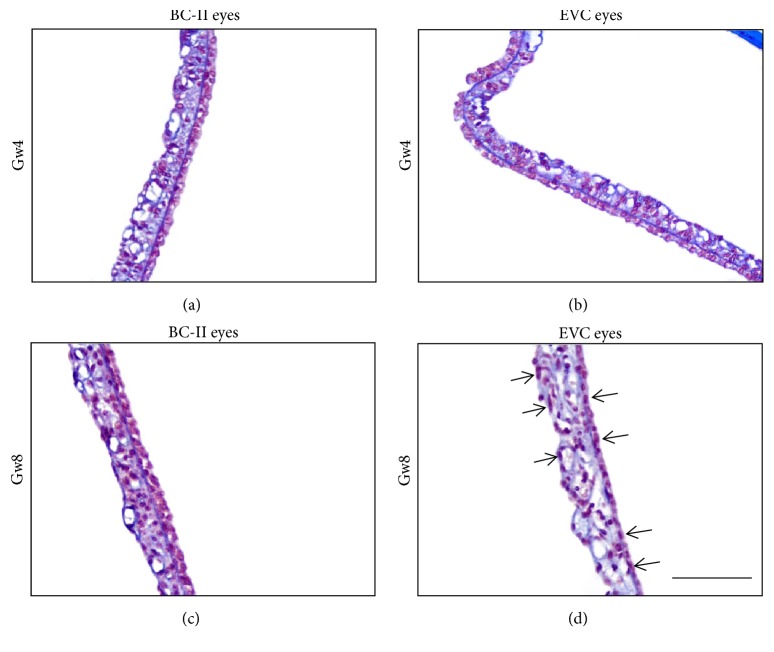
Typical section of rat iris. (a) Blank control-II eye (BC-II eye) in Gw4; (b) episcleral venous cauterization eye (EVC eye) in Gw4; (c) BC-II eye in Gw8; (d) EVC eye in Gw8. IPE in BC-II eyes shows a typical structure of two-layer epithelial cells. In EVC eyes (in both Gw4 and Gw8), IPE is obviously thinner, and the number of IPE cells has decreased. In Gw8, some cells were pressed from round balls into long strips (pointed by black arrows in (d)). The border between the stroma and IPE is not clear. Bar=100*μ*m.

Figure 5 shows the histological images of the OHT rats' iris in mid-periphery in Gw4 and Gw8. In Gw4, IPE was obviously thinner in EVC eyes, and the number of IPE cells in a 100-micron section had decreased (about 15 cells in EVC eyes compared with 20 cells in BC-II eyes). In Gw8, the border line between IPE and stroma could no longer be distinguished. IPE almost degenerated into a single layer. The number of IPE cells in a 100-micron section continued to decrease (about 10 cells in EVC eyes compared with 20 cells in BC-II eyes).

**Figure 6:**
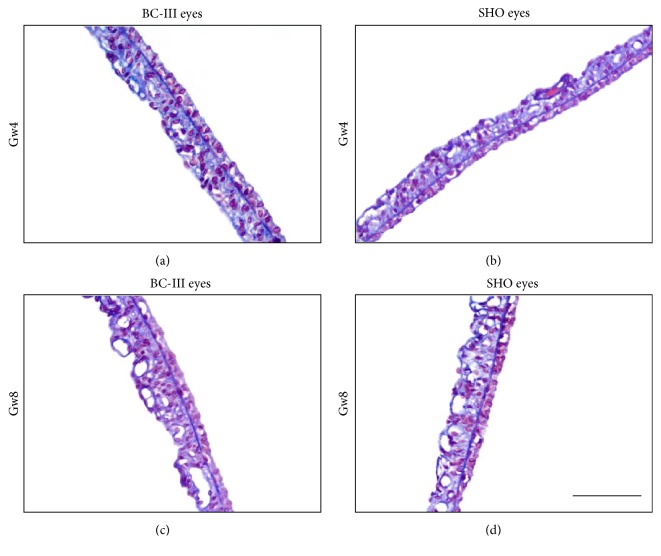
Typical section of rat iris. (a) Blank control-III eye (BC-III eye) in Gw4; (b) sham-operation eye (SHO eye) in Gw4; (c) BC-III eye in Gw8; (d) SHO eye in Gw8. Similar to BC-II eyes and BC-III eyes, the typical structure of two-layer epithelial cells of IPE can also be found in SHO eyes. Bar=100*μ*m.

Figure 6 shows the histological images of the sham-operation rat iris in mid-periphery in Gw4 and Gw8. Similar to BC-II eyes and BC-III eyes, the typical structure of two-layer epithelial cells also exists in SHO eyes.

### 3.3. Indentation

**Figure 7:**
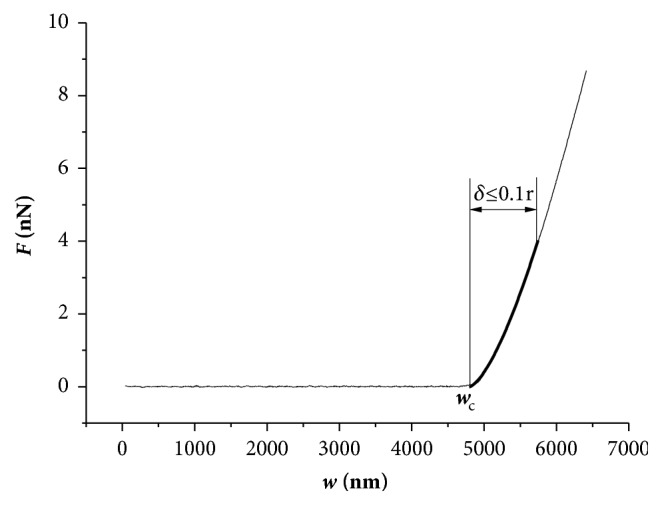
A typical indentation curve of **F**-**w**. The experimental data were fitted with Hertz equation to determine Young's modulus of iris. **w**_**c**_ was the value of **w** at the contact point, and **δ** was the depth of the indentation. The narrower curve was the experimental data, and the wider one was the regression curve.

**Figure 8:**
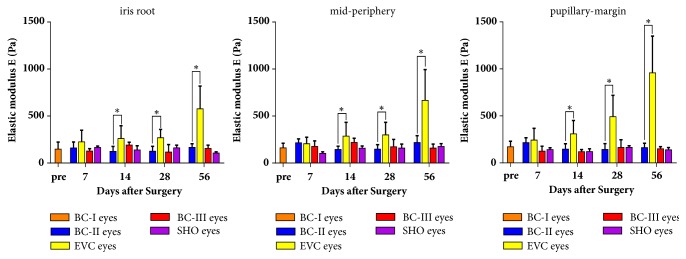
Elastic modulus of IPE in each region of each group. Data are shown as mean±sd. Comparisons are performed between EVC eyes and BC-II eyes and between SHO eyes and BC-III eyes (*∗*p <0.05). 28 days after surgery (Gw4) and 56 days after surgery (Gw8) in this figure do not include the rats used for histology.

Figure 7 shows a typical **F**-**w** curve. The experimental data were fitted by Hertz equation to determine the stiffness of IPE. Stiffness of IPE in each region of each group is shown in Figure 8. There was an overall growing tendency of IPE stiffness in EVC eyes. Meanwhile, IPE in EVC eyes was significantly stiffer than BC-II eyes in Gw2, Gw4, and Gw8 (in iris root, mid-periphery, and pupillary-margin, p<0.05). Regional differences in IPE stiffness of EVC eyes were also found in long duration of OHT (Gw4 and Gw8) but not in short duration of OHT (Gw1 and Gw2). IPE stiffness of EVC eyes in iris root, mid-periphery, and pupillary-margin was 270±85Pa, 299±128Pa, and 492±218Pa for Gw4 and 577±232Pa, 667±314Pa, and 958±375Pa for Gw8 (data are shown as mean±sd). IPE of EVC eyes in pupillary-margin was significantly stiffer than iris root in Gw4 and Gw8 (p<0.05). There was no significant difference in IPE stiffness between SHO eyes and BC-III eyes in Gw1, Gw2, Gw4, and Gw8.

## 4. Discussion

The current study provided the measurement of Young's modulus of IPE in rat eyes with OHT. It showed a growing trend from 1st week to 8th week after OHT induction. And from 2nd to 8th week, the IPE in EVC eyes was still stiffer than BC-II eyes (p<0.05). IPE of EVC eyes in pupillary-margin was significantly stiffer than iris root in Gw4 and Gw8 (p<0.05).

Compared with Mittag's method of OHT induction [22], our method reduced the number of injections of 5-Fu (only twice) and increased the interval between these two injections. Results indicated that our method could produce a long period (over 8 weeks) of moderate high intraocular pressure (about 30mmHg), a little lower than the result of Mittag (maybe because we reduced the number of injections of 5-Fu). To meet the welfare of animals (each animal has an unharmed eye at least), we did not inject 5-Fu in BC-II eyes of OHT rats but in SHO eyes of sham-operation rats. This method could also eliminate the possible interference of 5-Fu on intraocular pressure. The IOP of SHO eyes did not increase obviously, so the small elevation of IOP that occurred in the BC-II eyes (which was also shown in the result of Mittag) may be a neurological response but not because of injection of 5-Fu.

5-Fu is a kind of anticancer drug in clinical [26]. Subconjunctival injection of 5-Fu can inhibit the occurrence of new blood vessels, preventing the episcleral vein recanalization. As a cycle-specific agent, 5-Fu is only effective for cells with strong mitosis, such as the epithelial cells of the retina. IPE cells do not replicate rapidly in vivo, so IPE is supposed to be not sensitive to 5-Fu [27, 28]. On the other hand, there was basically no difference between SHO eyes and BC-III eyes in the sham-operation rats in IOP, histology, and IPE stiffness. So we believe that the 5-Fu dose we used in this experiment is safe and has little effect on the IPE.

Histological observation shows that, at 8th week after EVC, IPE is still continuous and uniform. So we believe that indentation tests are all performed on IPE. Some pigment cells become fusiform wherever in stroma or IPE in Gw8. This phenomenon suggests that pathologic pressure difference between the anterior and posterior chamber maybe appears in this rat model of chronic ocular hypertension. Besides, in Gw8, IPE is obviously thinner and even becomes a single layer. The number of pigment cells in the iris was significantly reduced. According to Sacca* et.al* [7, 8], excessive shedding of pigment epithelial cells may exceed the ability of trabecular endothelial cells to dissolve and accumulate in trabecular meshwork. Meanwhile, under chronic high intraocular pressure, the autophagy process of trabecular meshwork cells may be influenced. Both of these factors will lead to increased outflow resistance and further sustained high intraocular pressure. In our future work, we will try to observe the concentration of IPE in trabecular meshwork and explore whether the autophagy of trabecular cells is affected under high intraocular pressure. This work may give an explanation of sustained high IOP in mechanobiology.

Hertz model of contact is mainly based on two major assumptions: linear elasticity and infinite sample thickness. To maintain material linearity, the indentation *δ* should be less than 10% of sample thickness [25]. The thinnest IPE was in the EVC eye in Gw8 with thickness of 9*μ*m. IPE in other eyes were all thicker than 10*μ*m. So we chose data of *δ*≤1*μ*m for the calculation of elastic modulus.

Iris is a lamellar structure mainly composed of IPE and stroma (Figure 2). Our pretest showed that the stroma is also a kind of soft biomaterial, having almost the same Young's modulus as IPE. So the iris can be simplified as a kind of soft biomaterial when evaluating the influence of finite sample thickness. For correcting the finite thickness of the samples, according to Oncins [29] and Dimitriadis [25], the relationship between the applied force F and the indentation *δ* can be described by the corrected Hertz equation:

(2) F=169Er1/2δ3/21+0.884χ+0.781χ2+0.386χ3+0.0048χ4

where$\;\chi =\sqrt{r\delta }/h$, and r and h are the probe radius of curvature and the sample thickness, and [1 + 0.884**χ** + 0.781**χ**^2^ + 0.386**χ**^3^ + 0.0048**χ**^4^] is the correction coefficient. In this experiment, the petri dish is the substrate, and h is the thickness of iris which is about 50*μ*m, r=10*μ*m, and *δ*=1*μ*m. So **χ** in this research is about 0.063. And the correction coefficient is about 1.06. If the curve of F-*δ* is not fitted by the corrected Hertz model but the original Hertz model, the relative error of elastic modulus of IPE is about 6%. This relative error is less than the variable coefficient of the elastic modulus of IPE which varies from 20% to 60% in all kinds of eyes. Furthermore, we are concerned with the variation trend of the IPE stiffness with chronic high intraocular pressure. The relative error from the calculation of the elastic modulus of IPE can be neglected when it is far less than its variable coefficient of variation. Therefore using the original Hertz model is viable in our research.

It is also necessary to point out that the indentation of IPE in this research was performed in liquid phase. So jump-to-contact phenomenon which is mainly due to the van der Waals forces between the sample and the formation of a water meniscus when working in air operation at room conditions did not appear in the indentation curve of **F**-**w** in Figure 7[30].

IPE stiffness of blank-control eyes is 100~200Pa, which is basically the same as trabecular meshwork of rat measured by AFM [31]. We need also to acknowledge that rat IPE is softer than human, porcine, and bovine iris [21, 23, 32] which is more likely due to inherent interspecies differences and test method differences. Even so, this research can still give us some inspiration in the pathogenesis of glaucoma and provide basic data for subsequent research about AH flow in the posterior chamber.

In this study, IPE stiffness of EVC eyes shows a growing trend with the persistence of OHT. At 8th week after OHT induction, IPE of EVC eyes in Gw8 is obviously stiffer than BC-II eyes (about 4-fold) and BC-I eyes (about 4-fold). It has also been reported that iris [21] and trabecular meshwork [20] from glaucomatous human are stiffer than normal controls. These same trends reflect the important role of high intraocular pressure in the formation of glaucoma. Increased stiffness of IPE may be due to thicker inner IPE basement membranes under high intraocular which has been found in fine structure of glaucomatous donors' IPE [13]. Although the IOP in BC-II eyes was also increased, the smaller amplitude may not be enough to change the stiffness of IPE.

However, increased IPE stiffness may affect the flow field in turn. Previous work has verified that AH flow in posterior chamber is a noncontinuous and pulsatile flow [33], which may be more sensitive to the stiffness of wall than steady-flow. The stiffer IPE may increase pupillary blocking force [34] and the wall shear stress like other biological tissues such as airway [11] and intracranial aneurysms [35]. It would be possible to construct a bidirectional coupling model of AH and anterior segment [36] to illustrate the effect of IPE stiffness on the flow of the AH in the posterior chamber in our future work.

Eight-week duration of OHT for rats corresponds to about 6 years for human. The stiffness of IPE of blank-control eyes in each group has not changed significantly during this period. Previous clinical studies have shown that glaucoma is a disease that usually develops in the old rats. The rats in this research are in the young age, so the basically unchanged IPE stiffness of blank-control eyes during 8-week period fits our expectations. There also have been many researches about the age-related changes in the stiffness of ocular tissues such as cornea [37–39], sclera [40, 41], and lens [42, 43]. We will be able to perform indentation experiments on IPE of rats from youth to old age. The age correlation of IPE stiffness may be an inspiration for the high prevalence of glaucoma.

As a primary study, there are also some limitations in this study. First, it is important to note that the rats used for histology and for indentation were not the same. This may lead to dimensional error. A method to determine the dimensions of the iris components individually is necessary in our future work. Second, it should be noted that the indentation test is measuring an effective modulus, but not a true modulus. The IPE is viscoelastic and anisotropic, so using Hertz contact model to describe indentation is a simplification. Third, we have only reported that OHT leads to the IPE stiffness increasing gradually, due to lack of microcosmic explanation. It, however, has reported that suppression of the Wnt pathway plays an important role in the increasing stiffness of human trabecular meshwork cells [44]. Our future work will do some research of IPE on a molecular and cellular level. Fourth, iris also has another important part—stoma. For the small size of rat iris, it is hard to test the stoma and IPE by the same iris sample. Because this research is more concerned with AH flow in the posterior chamber, we choose to measure the stiffness of IPE.

## 5. Conclusion

The aim of this work did not take into consideration the homeostasis of the trabecular cells that also has relevance, but only the mechanistic aspects that however may play a role in the pupillary blocking force. During the duration of chronic high intraocular pressure, the stiffness of iris pigment epithelium increases gradually. Iris pigment epithelium in pupillary-margin was significantly stiffer than iris root in the eyes with 4-week and 8-week duration of chronic high intraocular pressure. The increased IPE stiffness may play a role in pupillary block and iris bombe. Further research on the effect of stiffer IPE on the flow field is necessary in the future work.

## Figures and Tables

**Table 1:** The number of each kind of rat prepared for indentation in each group.

Group	Rats	n	Eye	Full name and short name of the eye
Gw0	Blank-control	3	Left	Blank-control eye (BC-I eye)
			Right	Blank-control eye (BC-I eye)

Gw1	OHT	6	Left	Blank-control eye (BC-II eye)
			Right	Episcleral venous cauterization eye (EVC eye)
	sham-operation	2	Left	Blank-control eye (BC-III eye)
			Right	Sham-operation eye (SHO eye)

Gw2	OHT	6	Left	Blank-control eye (BC-II eye)
			Right	Episcleral venous cauterization eye (EVC eye)
	sham-operation	2	Left	Blank-control eye (BC-III eye)
			Right	Sham-operation eye (SHO eye)

Gw4	OHT	6	Left	Blank-control eye (BC-II eye)
			Right	Episcleral venous cauterization eye (EVC eye)
	sham-operation	2	Left	Blank-control eye (BC-III eye)
			Right	Sham-operation eye (SHO eye)

Gw8	OHT	6	Left	Blank-control eye (BC-II eye)
			Right	Episcleral venous cauterization eye (EVC eye)
	sham-operation	2	Left	Blank-control eye (BC-III eye)
			Right	Sham-operation eye (SHO eye)

## Data Availability

The data used to support the findings of this study are available from the corresponding author upon request.

## Abbreviations

IPE: Iris pigment epithelium
OHT: Ocular hypertension
EVC: Episcleral venous cauterization
AFM: Atomic force microscope
IOP: Intraocular pressure
AH: Aqueous humor
SHO eye: Sham-operation eye
BC-I eye: Blank-control eye (left/right eye) of Blank-control rat in Gw0
BC-II eye: Blank-control eye (left eye) of OHT rat in Gw1, Gw2, Gw4, and Gw8
BC-III eye: Blank-control eye (left eye) of Sham-operation rat in Gw1, Gw2, Gw4, and Gw8
5-Fu: 5-fluorouracil
PBS: Phosphate buffered saline.
